# Conflict-Elicited Negative Evaluations of Neutral Stimuli: Testing Overt Responses and Stimulus-Frequency Differences as Critical Side Conditions

**DOI:** 10.3389/fpsyg.2019.02204

**Published:** 2019-10-15

**Authors:** Florian Goller, Alexandra Kroiss, Ulrich Ansorge

**Affiliations:** ^1^Faculty of Psychology, University of Vienna, Vienna, Austria; ^2^Department of Linguistics, University of Vienna, Vienna, Austria; ^3^Vienna Cognitive Science Hub, University of Vienna, Vienna, Austria

**Keywords:** Stroop task, fluency, misattributions of affect, conflict, stimulus frequency

## Abstract

Prior research has shown that a stimulus signaling a conflict (such as an incongruent Stroop stimulus) as a prime can elicit more negative evaluations of an otherwise neutral and unrelated stimulus as a target. Yet, there are many side conditions that could at least partly be responsible for such effects like the frequencies of congruent and conflicting stimuli or overt responses to the conflicting stimuli. Here, we tested the influences of stimulus frequencies and overt responses on the strength of this priming effect. In four experiments, we demonstrate that overt responses in-between prime and target do not delete the conflict-elicited evaluation effect (Experiments 1a vs. 1b), while an overall higher frequency of conflicting trials (Experiment 2a) and an overall lower frequency of congruent trials (Experiment 3) can both abolish the priming effect. In contrast, a higher frequency of specific conflicting conditions was ineffective (Experiment 2b). Together, our results confirm that conflict is indeed the origin of the priming of negative evaluations.

## Introduction

In general, evaluations of stimuli as positive or negative are important for a variety of basic human functions, such as learning by reinforcement (Sutton and Barto, 1998), optimal decisions (Lewin, 1926; Janis and Mann, 1977), or general wellbeing (Seligman and Csikszentmihalyi, 2000). As an example, think of decisions, where usually humans strive to choose options leading to positive results. Typically, evaluations of stimuli are a consequence of some properties of the evaluated stimuli themselves, such as their taste or their beauty. Importantly, however, research has identified diverse sources of such evaluations that have little to do with the stimulus itself but rather with the degree of effort exerted in cognitive performance temporally close to the stimulus in question. This is the topic of the present investigation as we will explain next.

Here, we studied three such potential influences and their interplay. First, cognitive conflict elicited by incongruent stimuli. Such conflict in and by itself is negative, as it corresponds to a demanding task. Such conflict is, therefore, one factor that potentially prompts negative evaluations of stimuli temporally close but actually unrelated to the task at hand (Fritz and Dreisbach, 2013; Damen et al., 2018). Second, however, correct responses given to conflicting stimuli can be felt as particularly gratifying because of the higher pleasure one takes in successfully mastering challenging compared to easier situations (cf. Schouppe et al., 2015; Ivanchei et al., 2018). Third, the mere frequency of conflict or of particular (conflicting) stimulus configurations can facilitate processing and could also be experienced as (more) positive (cf. Winkielman et al., 2003). These are influences with the potential to foster a positive evaluation of an otherwise neutral stimulus in close temporal vicinity to task performance.

Remarkably, although it is immediately obvious that these factors could interact, how these three influences work in concert is unknown. For example, it is clear from the above that conflict itself versus a correct response to a conflicting stimulus could have counteracting influences on the final evaluation of a neutral stimulus (Ivanchei et al., 2018). In fact, slower responses in incongruent than congruent conditions might also revert the influence of conflict on evaluations merely through the longer response times and, thus, the more time that passes between conflicting stimulus and to-be-evaluated stimulus (cf. Fritz and Dreisbach, 2015; but see Damen et al., 2018). Yet, assigning and requiring alternative responses to conflicting stimuli could in principle also strengthen the felt conflict and, hence, the negative evaluation of a neutral stimulus (but see Damen et al., 2018). Take the flanker task as an example, where a target letter (e.g., an *A*) is presented at screen center together with sensory conflicting stimuli as irrelevant flankers (e.g., *XAX*). Interference by the flanker letters increases if alternative responses are required for target versus flankers (e.g., a left-hand response to the *X* if used as a target and a right-hand response to the *A* as a target) relative to requiring the same response for target and flankers (Eriksen and Eriksen, 1974). As such, response conflict on top of sensory conflict should sometimes elicit more experienced conflict than mere sensory conflict. Therefore, despite the relief or pride a participant might feel by having given a correct response to such a conflicting stimulus, response conflict might still create a net negative influence on the evaluation of a subsequently presented neutral stimulus. For example, response times of correct responses to conflicting stimulus configurations are typically slower than response times of correct responses to non-conflicting stimulus configurations. At least if noticed, this could also lead to negative feelings on the side of the participant such as disappointment or frustration about one’s own performance. In addition, introducing the third factor mentioned above into the hypotheses, frequency manipulations influence the degree of conflict by a stimulus or how much one relies on the conflicting source in the first place. For example, in situations where a lot of conflict pertains on average, participants take greater care not to process the conflicting stimuli. In contrast, in situations in which correlations of relevant stimuli and irrelevant conflicting stimuli provide occasional advantages, participants could more willingly incorporate the conflicting stimulus into their task performance (e.g., Melara and Algom, 2003). Before we turn to the current experiments that we conducted to answer such questions about the interplay of different facets of task performance on evaluations of temporally close neutral stimuli, we will first review existing evidence on this question.

To start with, in line with the first principle mentioned above, cognitive conflict can elicit negative evaluations, even if these evaluations concern other stimuli than those creating the cognitive conflict (Fritz and Dreisbach, 2013, 2015). In contrast to cognitive congruency (e.g., a similarity of meaning of relevant and irrelevant stimulus), cognitive conflict elicits negative affective responses (Botvinick, 2007; van Steenbergen et al., 2009; Dreisbach and Fischer, 2012, 2015, 2016; Chetverikov and Kristjánsson, 2016; for a review, see Inzlicht et al., 2015). For example, Stroop incongruency (cf. Stroop, 1935; for a review, see MacLeod, 1991; MacLeod and MacDonald, 2000) between print color and color word meaning presented as a prime facilitates the processing of subsequently presented negative targets (Dreisbach and Fischer, 2012; Ligeza and Wyczesany, 2017). This is in comparison to congruency between print color and color word meaning that would facilitate the processing of positive targets instead. Such Stroop incongruency can even elicit negative evaluations of otherwise neutral objects, like neutral Chinese characters that are unknown to non-Chinese reading participants (Fritz and Dreisbach, 2013, 2015). These effects are possibly due to priming: The negative evaluation elicited by the conflict prevails in memory until the next stimulus is encountered and, thereby, the negative evaluation of the conflict primes a negative judgment about a following but otherwise neutral stimulus (cf. Hermans et al., 1994).

However, an important factor for whether conflict promotes a negative evaluation of a task-unrelated stimulus could be whether or not a correct response is given to the conflicting stimulus and before the task-unrelated and to-be evaluated neutral stimulus. It has been shown that successful task performance like giving the correct response to a conflicting stimulus can trigger a positive feeling (Schouppe et al., 2015). As a consequence, a task that has been experienced as negative before it has been mastered because of its challenge or demand could give way to a positive appraisal after being solved and lead to feelings such as pride or relief. In line with this observation, conflict-elicited negative evaluations of otherwise neutral stimuli are sometimes observed where no response is given between conflicting stimulus and to-be evaluated neutral Chinese character (Fritz and Dreisbach, 2013), but the same conflict-elicited negative evaluations are seemingly abolished where a correct response is given to a conflicting stimulus and before the evaluation of a neutral stimulus (Goller et al., 2017). Yet, theoretically, the opposite results are also possible: Responses could boost priming of subsequent negative evaluations if a response conflict highlights the incongruency between stimuli even further. In this context, one should note that there were differences between the past studies in question, such as a different type of stimulus conflict (between colors and meanings in Fritz and Dreisbach, 2013; and between prime and target shapes in Goller et al., 2017) and a different task (a Stroop task in Fritz and Dreisbach, 2013; and a priming task in Goller et al., 2017). Such influences could be responsible for different results in the respective studies, too. In other words, the question which role overt responses to conflicting stimuli play for the evaluation of the neutral stimuli has not been finally settled so far (e.g., Damen et al., 2018 vs. Schouppe et al., 2015).

Here, we studied once more if overt responses to a conflicting stimulus can abolish the conflict-elicited priming of a subsequent negative evaluation of a neutral stimulus or whether these responses may even boost priming of a negative evaluation of a subsequent stimulus where alternative responses highlight the stimulus incongruency. These possibilities were tested by an exact replication of the Stroop procedure of Fritz and Dreisbach (2013) in Experiment 1a and by its comparison to a version of the task in Experiment 1b where a response was given to the conflicting or congruent stimuli prior to the evaluation of a neutral Chinese character. In addition, we used various manipulations to alter the degree of conflict experienced to demonstrate that it is indeed the conflict that drives the priming effect. We varied the frequency of conflicting relative to congruent conditions in general (Experiment 2a) and of specific conflicting conditions in particular (Experiment 2b) and expected lower conflict and less priming of negative evaluations for higher frequencies of (particular) conflicting conditions. Finally, we also varied the degree to which alternative stimuli (here: color meanings) were indeed associated with an alternative response (Experiments 1a and 3). We expected that whatever modulating effect a response in-between conflicting stimulus and to-be-evaluated stimulus might have (i.e., a diminishing or a supporting effect) should be present where conflicting stimuli were associated with alternative responses but not or less so where conflicting stimuli were motor congruent (Experiment 1a) or response-irrelevant (Experiment 3). For example, an incongruent Stroop stimulus such as the word *BLUE* in green was presented in an experiment in which the colors blue and green required alternative responses (i.e., in motor incongruent conditions). In this case, conflict-elicited evaluation effects that depend upon motor conflict should be stronger than in situations in which the same incongruent stimulus would be motor congruent because blue and green required the same response from the participants.

## Experiment 1A

Experiments 1a and 1b were exact replications of the procedure of Fritz and Dreisbach (2013), with the exception that an overt response to the Stroop stimuli was required in Experiment 1b only. Past research that compared the influence of overt responses on conflict-elicited evaluations in the Stroop task found no such influences (Damen et al., 2018). However, compared to the original study of Fritz and Dreisbach (2013), Damen et al. (2018) used relatively long intervals between conflicting and to-be-evaluated stimuli. As this interval can have an influence on conflict-elicited evaluations (Fritz and Dreisbach, 2015), we wanted to replicate the findings with the shorter interval, at least in the conditions without a response, used by Fritz and Dreisbach (2013).

### Materials and Methods

#### Participants

Forty-eight participants (32 females and 16 males, *M*_*Age*_: 22.31 years, *SD*_*Age*_: 5.34 years) from the subject pool of the University of Vienna were tested. A power analysis yielded a sample size of 24 participants as sufficient. We calculated the sample sizes for all experiments by using G^∗^Power (Faul et al., 2009) under the assumption of a medium effect size (Cohens’ *d* = 0.6) and a statistical power of 90%. Our calculated sample size was also similar to the sample size in the original study by Fritz and Dreisbach (2013). In Experiment 1, we doubled the calculated sample size to control for effects of the order/sequence of experiments (first Experiment 1a then Experiment 1b versus first Experiment 1b then Experiment 1a). Here and in all following experiments, participants received course credit, were right-handed, had normal or corrected-to-normal vision, and reported no prior experience with Chinese characters. Prior to all experiments, all participants gave written consent and were informed that participation and data collection were fully anonymous. Participants could withdraw at any time during the experiment without any further consequences. All studies were conducted in accordance with the Declaration of Helsinki (revised, 1983) and the guidelines of the Faculty of Psychology, University of Vienna. We further followed the Austrian Universities Act, 2002 (UG2002) – which was active at the time of the experiments – which required only medical universities to appoint ethics committees for clinical testing, and application of medical methods and applied medical research. Therefore, no additional ethical approval was sought.

#### Apparatus and Stimuli

Stimuli were presented on a 19″ LCD Monitor (Acer B 193) with an aspect ratio of 4:3 and a resolution of 1,024 × 768 pixels at a vertical refresh rate of 75 Hz. Viewing distance was kept stable at 57 cm by a chin and forehead rest. Manual responses were recorded as keypresses of the left and right index fingers on a standard computer keyboard. The experiment was programmed and controlled using Matlab 7.7.0 (The MathWorks Inc., MA, United States) and the Psychophysics Toolbox (Brainard, 1997; Pelli, 1997).

All stimuli were presented against a gray (CIE Lab: 112.2 cd/m^2^; −3.9/−34.2) background. Chinese characters (8° × 8°) and the central fixation point (0.5°) were black (0.7 cd/m^2^; 0.0/−0.3) and were presented at the center of the screen. In total, 200 Chinese characters were randomly chosen from an online English–Chinese dictionary^1^. Stroop words (i.e., the German words for BLUE, GREEN, YELLOW, RED, and PURPLE) were written in Courier New bold (23 pt.), each letter subtending a visual angle of approximately 0.7° × 0.9° and were presented in red (73 cd/m^2^; 98.1/97.0), blue (24.8 cd/m^2^; 48.1/−96.0), green (105.5 cd/m^2^; −109.0/81.3), yellow (130.7 cd/m^2^; −31.1/109.6), or purple (48.1 cd/m^2^; 77.4/−64.8).

#### Procedure

**FIGURE 1:**
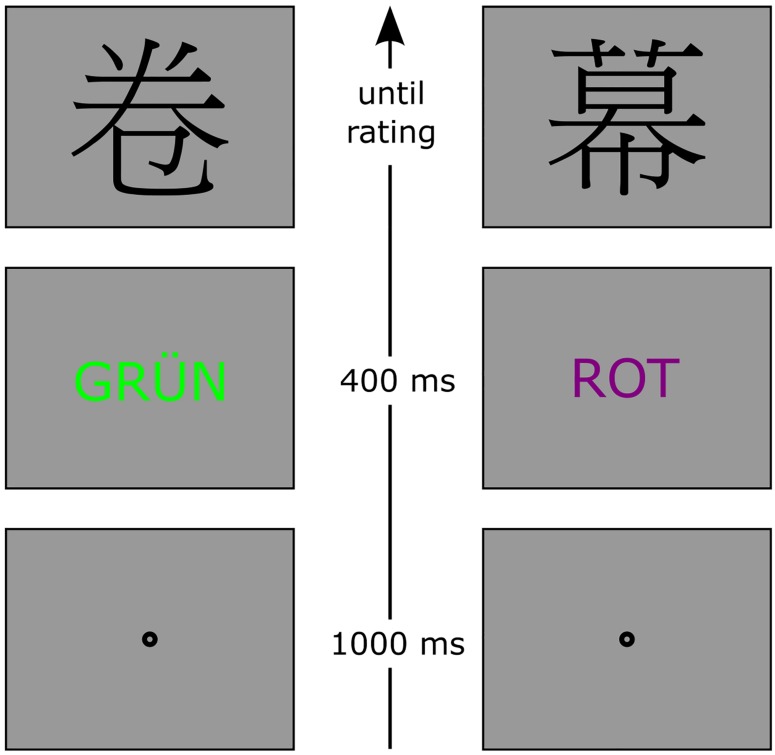
Procedure of Experiment 1a with time flowing from bottom to top. If the Stroop stimulus (second screen) was of purple color or the word PURPLE, participants had to press the space bar instead of evaluating the character. The character was shown nonetheless. The figure is not drawn to scale. GRÜN, German for GREEN; ROT, German for RED.

All participants were tested in Experiments 1a and 1b (see below) in a single experimental session. The order of experiments (first 1a then 1b; first 1b then 1a) was counterbalanced across participants. Experiments 1a and 1b were interrupted by a self-paced break. In Experiment 1a, we replicated the exact design of Fritz and Dreisbach (2013) (see also Figure 1). Participants had to judge the Chinese characters as either negative (left button) or positive (right button). This assignment was kept constant within and across participants. Participants were instructed to give a fast and intuitive evaluative judgment and not to rethink their decision. Processing of the Stroop stimuli was ensured through catch trials: Whenever a word was printed in purple or was the German word for PURPLE, participants had to press the space bar instead of evaluating the Chinese character. In these trials, a Chinese character was still shown, but no judgment was required. Catch trials were always Stroop incongruent.

After a fixation display of 1 s, the Stroop stimulus was displayed for 400 ms, immediately followed by the Chinese character, which was displayed until the evaluation or the pressing of the space bar (in catch trials only). An erroneous response (pressing the space bar in a non-catch trial or pressing an evaluation key in a catch trial) triggered visual error feedback that appeared at screen center.

The experiment consisted of 60 sensory congruent (stimulus color = color word meaning), 60 sensory incongruent or conflicting (stimulus color ≠ color word meaning), and 24 catch trials. In incongruent trials, each of the 12 possible combinations of Stroop word and Stroop color was presented equally often. The characters were randomly assigned to the experimental conditions and varied between participants. Participants received 20 practice trials to familiarize themselves with the task. All conditions were shown in a random sequence. Together with instructions and debriefing, Experiments 1a and 1b took about 30–40 min in total.

### Results

#### Statistical Analysis

For the statistical analyses, we used (generalized) linear mixed models due to a logistic regression yields more statistical power for dichotomous data than any data transformation (Warton and Hui, 2011). Furthermore, we are able to account for the random variance caused by the (in terms of, e.g., visual complexity) different Chinese characters we used in our experiments. For our continuous data (reaction times, RTs) we used a linear mixed model, for the analysis of the dichotomous data [evaluations, error rates (ERs)] we used a generalized linear mixed model with a binomial (logit) distribution as a link function. All models were computed using R 3.5.1 (R Core Team, 2018) and the lme4 package 1.1-18-1 (Bates et al., 2015). Calculation of the *p*-values was carried out via the afex package 0.21-2 (Singmann et al., 2018), by either using likelihood-ratio tests (dichotomous data) or a Satterthwaite approximation (continuous data). In each model, we included random slopes for the different Chinese characters (items).

One problem is that a non-significant *p*-value does not inform us about whether truly no effect was found or our test was insensitive to the effect (see Dienes, 2014). To resolve this problem, we additionally conducted Bayesian factor (BF) analysis of critical comparisons that yielded null results (Rouder et al., 2009). Here we reported the scaled JZS Bayes factor values. They indicate the relation between the probabilities of the data being in favor of the null relative to being in favor of the alternative hypothesis (or vice versa). On the basis of Jeffreys (1961) convention, a Bayes factor >3.00 is considered as substantial evidence in favor of the null or the alternative hypothesis accordingly (Dienes, 2011).

#### Evaluations

The mean ER on catch trials was 9.03%, *SD* = 5.20%. Catch trials were omitted from the analysis. From the remaining data, all incorrect reactions (i.e., pressing the spacebar instead of one of the evaluation keys; 0.59%) were removed. To test for a general bias toward positive or negative evaluations, we compared the proportions of negative judgments (across conditions) to the null hypothesis of unbiased evaluations corresponding to 50%. The overall proportions of evaluations showed no tendency toward a positive or negative bias (51.27%), *t*(47) = 0.61, *p* < 0.249, *d* = 0.09, BF = 4.03 in favor of the null hypothesis.

For the analysis of the evaluations, we fitted the evaluations to a generalized linear mixed model with sensory congruency and experiment order as fixed factors. By-participants random intercepts and slopes for sensory congruency (without their correlation), as well as random intercepts by Chinese characters were used. Sensory congruency yielded a significant effect, χ^2^(1) = 8.60, *p* = 0.003, indicating that evaluations were significantly more negative in the sensory incongruent (estimate = −0.23, *SE* = 015) than in the sensory congruent condition (estimate = 0.35, *SE* = 0.14). Neither the effect of experiment order nor the interaction between experiment order and sensory congruency was significant, all χ^2^ < 3.06, all *p* > 0.080. See also Table 1, upper row.

**TABLE 1:** The percentages of negative evaluations as a function of sensory congruency (columns) in Experiments 1a, 2a, and 2b (rows).

	**Sensory congruency**	
	**Congruent (%)**	**Incongruent (%)**
Experiment 1a	38.13	54.54
Experiment 2a	44.76	49.14
Experiment 2b	32.70	59.20

#### Reaction Times – Evaluation

To test if these results are moderated by the time participants took to rate the Chinese characters, we subjected the centered RTs [RT – mean(RT)] to a model with the fixed factors sensory congruency and evaluation and their interaction. Random intercepts and slopes for this interaction by participant, as well as random by Chinese characters intercepts were used. No significant result was found, all *F* < 1.26, all *p* > 0.249.

## Experiment 1B

### Materials and Methods

#### Participants

The same 48 participants as in Experiment 1a participated. We excluded one participant due to an ER in the Stroop classification responses of 47%. Note that in contrast to Experiment 1a, now responses to the colors of the Stroop stimuli were required, see below.

#### Apparatus, Stimuli, and Procedure

**FIGURE 2:**
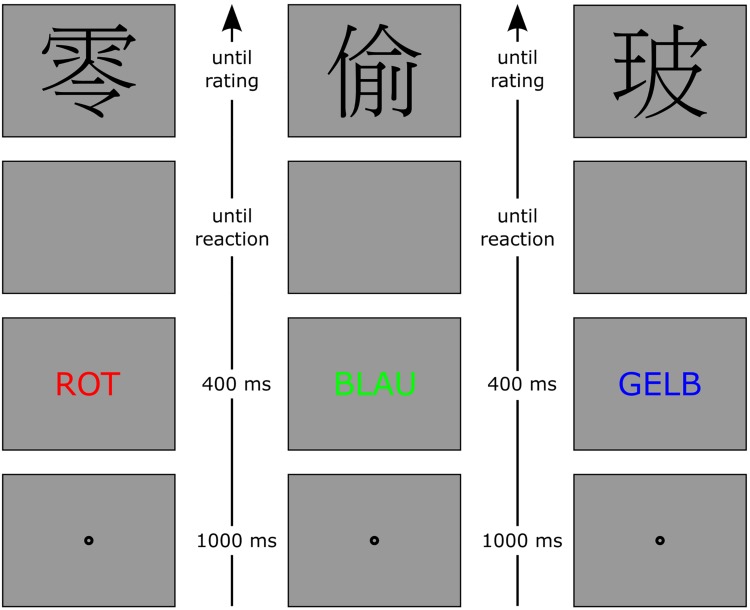
Procedure of Experiment 1b with time flowing from bottom to top. After the presentation of the Stroop stimulus, participants had to classify the Stroop color as either warm or cold. The Stroop stimuli were presented for 400 ms followed by a blank screen. The depicted conditions are motor and sensory congruent on the left (word color is congruent to color word and mapped on the same key), motor congruent and sensory incongruent in the middle (word color is incongruent to color word but the colors are still mapped on the same key), and motor and sensory incongruent on the right (word color is incongruent to color word and the colors are mapped to different keys). The figure is not drawn to scale. ROT, German word for RED; BLAU, German word for BLUE; GELB, German word for YELLOW. After the classification of the Stroop stimulus, a Chinese character had to be evaluated.

Experiment 1b was a replication of Experiment 1a with two notable differences in regards to the procedure: First, participants had to give an overt response to classify the color of the Stroop stimulus before evaluating the Chinese character (Figure 2). The color had to be classified as warm (red/yellow) or cold (blue/green). Each Stroop stimulus was presented for 400 ms, followed by a blank screen until the participant’s color classification was registered. Participants could give their answers immediately after the onset of the Stroop stimulus. The Stroop word PURPLE, as well as the Stroop color purple, was no longer used. Stimulus-to-response mapping was constant within but counterbalanced across participants. Participants used the same keys for the classification (warm/cold) and the evaluation (negative/positive). Experiment 1b consisted of 128 trials which were split into 64 motor conflicting (which were also sensory incongruent, e.g., the word BLUE in red) and 64 motor congruent trials (half of which were also sensory congruent, e.g., the word BLUE in blue, and half were sensory incongruent, e.g., the word BLUE in green). To be exact, the motor congruent trials consisted of 32 sensory congruent (stimulus color = color meaning) and 32 sensory incongruent or conflicting trials (stimulus color ≠ color meaning). Thus, sensory and motoric congruency versus incongruency could not be orthogonally crossed because there is no motor conflicting and sensory congruent condition.

### Results

#### Reaction Times – Stroop Classification

We removed all trials with erroneous responses (8.29%) in the Stroop color classification task and all trials with an RT <100 ms or >2.5 *SD*s of the mean RT of the individual participant per condition (2.60%). The remaining RTs were centered and subjected to a linear mixed model with the fixed factors congruency (motor congruent/sensory congruent; motor congruent/sensory incongruent; motor incongruent/sensory incongruent) and experiment order (1a–1b; 1b–1a). Random intercepts and slopes for congruency by participant, as well as random intercepts by Chinese characters were used. A significant effect of congruency, *F*(2,58.65) = 21.63, *p* < 0.001, was followed up with pairwise Holm-corrected comparisons of the estimated marginal means of the model. These yielded significant differences between all pairs, all *t* > |3.89|, all *p* < 0.001. RT increased from motor congruent/sensory congruent conditions to motor congruent/sensory incongruent conditions and to motor incongruent/sensory incongruent conditions (Table 2). No other effects or interaction of the model were significant, all *F* < 0.89, all *p* > 0.249.

**TABLE 2:** Reaction times (in ms) of the Stroop task as a function of sensory congruency (columns) and motor-congruence/motor-incongruence (rows) in Experiment 1b.

	**Sensory congruency**	
	**Congruent (ms)**	**Incongruent (ms)**
Motor congruent	677	711
Motor incongruent	–	741

#### Error Rates – Stroop Classification

The classifications of the Stroop stimuli (correct vs. false) were subjected to a generalized linear mixed model similar to the RT, but without random slopes for congruency by participant because the model did not converge otherwise. Contrasts were set to grand mean ERs across all levels of the independent variable congruency. While the estimated overall mean was significantly different from zero (estimate = 2.70, *SE* = 0.14, *z* = 20.00, *p* < 0.001), none of the different levels of congruency differed from this mean (all estimates < |0.15|, all *p* > 0.249), indicating roughly similar ERs across all conditions. No other effects or interaction of the model were significant, all estimates < |0.11|, all *p* > 0.130.

#### Evaluations

This analysis was based on the same data as the analysis of the RT in the Stroop task (all errors and RT outliers were excluded). We found an overall tendency for more positive evaluations (55.13% positive vs. 44.87% negative evaluations), *t*(44) = 3.62, *p* < 0.001, *d* = 0.53. We fitted the evaluations to a generalized linear mixed model with congruency (motor congruent/sensory congruent; motor congruent/sensory incongruent; motor incongruent/sensory incongruent) and experiment order as fixed factors. By-participants random intercepts and slopes for congruency, as well as random intercepts by Chinese characters were used. Contrasts were set to grand mean across all levels of the variable congruency. In relation to this overall mean (estimate = 0.29, *SE* = 0.07, *z* = 4.43, *p* < 0.001), the motor incongruent/sensory incongruent condition showed a significantly higher probability of a negative evaluation (estimate = −0.23, *SE* = 0.07, *z* = −3.44, *p* < 0.001) and the motor congruent/sensory congruent condition showed a significantly lower probability of a negative evaluation (estimate = 0.12, *SE* = 0.04, *z* = 2.80, *p* = 0.005). Neither the effect of experiment order nor the interaction between experiment order and sensory congruency were significant, all χ^2^ < 2.44, all *p* > 0.120. See also Table 3.

**TABLE 3:** The percentages of negative evaluations as a function of sensory congruency (columns) and motor-congruence/motor-incongruence (rows) in Experiment 1b.

	**Sensory congruency**	
	**Congruent (%)**	**Incongruent (%)**
Motor congruent	38.19	38.98
Motor incongruent	–	48.37

#### Reaction Times – Evaluation

Analogous to Experiment 1a, we subjected the centered evaluation times to a linear mixed model with the fixed factors congruency and evaluation (positive/negative) and their interaction. Random intercepts and slopes for this interaction by participant, as well as random intercepts by Chinese characters were used. No significant result was found, all *F* < 1.49, all *p* > 0.230.

#### Consistency of Evaluations

The Chinese characters each participant had to rate were randomly drawn from a pool of 200 available characters. However, there was a considerable number of Chinese characters that were rated by a participant both in Experiments 1a and 1b. Therefore, the same Chinese character could have been presented to the same participant in different congruency conditions in Experiment 1a than in 1b. To assess whether the evaluations were consistent between Experiments 1a and 1b, we selected all cases where a participant rated the same character twice (across all participants 5,737 cases) and calculated the correlation between these two evaluations. Indeed, we found a small but significant positive correlation: *r*(5,737) = 0.08, *p* < 0.001.

#### Key Repetitions

The classification of the Stroop stimulus and the evaluation of the Chinese character were mapped on the same keys. By consequence, participants were sometimes required to press the same key twice. This could have induced the response strategy to frequently press the same key. In Experiment 1b, the proportion of trials where participants pressed the same key twice was 53.44% which was not significantly different from 50%, *t*(47) = 1.98, *p* = 0.064, *d* = 0.34. This result could potentially mean that participants used such a response strategy. To ensure that this did not affect our overall results (e.g., diminished a true effect of Stroop congruency on evaluations), we fitted the evaluations to a model with the fixed factor key repetition (key alternation; key repetition). In relation to the overall mean (estimate = 0.19, *SE* = 0.07, *z* = 2.66, *p* = 0.008), key repetition did not yield a significant effect, estimate = 0.07, *SE* = 0.06, *z* = 1.10, *p* > 0.249, BF = 4.70 in favor of the null hypothesis.

### Discussion

After Experiment 1, it is clear that the overt response did not eliminate or did not reverse the priming effects of conflicting stimuli on evaluations of subsequently presented neutral Chinese characters. It is true that the influence of sensory conflict alone that was present in Experiment 1a was gone in Experiment 1b. However, this influence probably was modified by assigning a congruent response meaning to these only sensory incongruent stimuli and it was numerically still in the predicted direction: More negative evaluations were elicited by sensory conflicting than by sensory congruent conditions. Importantly, this difference was much stronger for motor incongruent conditions. Thus, the results of Experiment 1b suggested that by assigning the same response to different stimuli (here: its features or dimensions) this shared motor meaning can diminish the influences of mere sensory conflict on negative evaluations. However, the results are at variance with the assumption that overt responses to the incongruent stimuli could have eliminated the priming effect of conflict on evaluations of the neutral Chinese characters. One reason could be that the participants experienced more negative feelings (e.g., frustration) about their slower responses in incongruent (conflicting) compared to congruent trials than feelings of pride or relief after managing to correctly perform on a conflicting trial.

Besides these major findings, it can be ruled out that specifics of our task, such as a tendency to simply press the same button twice – once for the target of the Stroop task and once for the evaluation of the Chinese character – accounted for the findings in Experiment 1b. These button press repetitions and button press changes from Stroop stimulus to Chinese character were about equally likely and did not affect (i.e., were not inversely related to) the evaluations. The same is true of an influence of consistency of evaluations of Chinese characters across Experiments 1a and 1b. Although there was a slight correlation between Chinese character evaluations, this correlation was small and not all characters were rated twice. Hence, our method was sufficiently sensitive to potential differences in conflict-elicited evaluations between conditions with and without overt responses.

## Experiment 2A

Following Experiment 1, we manipulated the degree of conflict to understand if higher frequencies of incongruent stimulus configurations diminished the priming of a negative evaluation. Experiment 2a used the exact same procedure as Fritz and Dreisbach (2013) in that participants had to give no response to the Stroop stimuli prior to the character evaluations. However, the frequency of the different congruent and incongruent stimuli was manipulated. In Experiment 2a, incongruent stimuli were three times more likely than congruent stimuli. To the extent that congruent stimuli invite the use of the irrelevant word meaning (cf. Logan and Zbrodoff, 1979), the present Experiment 2a discourages this use and, by implication, the degree of conflict in incongruent trials. As a consequence, we expected the priming of negative evaluations to drop. Another factor that could diminish the conflict in Experiment 2a was the mere frequency with which each specific incongruent stimulus was presented. Each specific incongruent stimulus was presented in 25% of all trials. Thus, the frequencies were much higher than that of each specific conflicting stimulus in Experiment 1 (10% in Experiment 1a and 12.5% in Experiment 1b). Higher frequencies of each specific incongruent Stroop stimulus could also diminish conflict by increased felt fluency (cf. Winkielman et al., 2003).

### Materials and Methods

#### Participants

Twenty-four participants were tested, but one participant was *post hoc* excluded due to >20% of errors. The remaining sample contained 21 females and 2 males, *M*_*Age*_: 21.09 years, *SD*_*Age*_: 5.48 years.

#### Apparatus, Stimuli, and Procedure

Experiment 2 was similar to Fritz and Dreisbach (2013) and to the current Experiment 1a, with the notable exception that each specific sensory signal (i.e., each color/color-word combination) was shown equally often. There are three times as many possibilities to create an incongruent Stroop (RED in green, RED in yellow, RED in blue) compared to a congruent Stroop (RED in red) stimulus. In this experiment, we increased the number of incongruent trials to make each sensory stimulus (e.g., RED in green) appear equally often. By consequence, there were now three times more incongruent than congruent trials.

### Results

#### Evaluations

The mean ER was 1.65%. Catch trials and all trials with an incorrect reaction were omitted from the analysis. To test for a general bias toward positive or negative evaluations, we tested the percentages of negative judgments (across conditions) to the null hypothesis of 50%. We found no overall tendency (53.05% positive vs. 46.95% negative evaluations), *t*(22) = 0.75, *p* > 0.249, *d* = 0.22, BF = 4.08 in favor of the null hypothesis. We fitted the evaluations to a generalized linear mixed model with sensory congruency as a fixed factor. By-participants random intercepts and slopes for sensory congruency, as well as random intercepts by Chinese characters were used. No significant effect of sensory congruency was found, χ^2^(1) = 1.21, *p* = 0.270, BF = 2.76 in favor of the null hypothesis, indicating that evaluations did not differ between the sensory incongruent (estimate = −0.05, *SE* = 0.24) and the sensory congruent condition (estimate = 0.16, *SE* = 0.29). See also Table 1, middle row.

#### Reaction Times – Evaluation

Again, the time it took participants to rate the Chinese characters were centered and subjected to a linear mixed model, with evaluation (positive/negative) and sensory congruency (congruent/incongruent) as fixed effects and the same random effects structure as in Experiment 1a. No significant effects were found, all non-significant *F* < 0.81 all *p* > 0.249.

### Discussion

As predicted, the results of Experiment 2a showed that an increased frequency of incongruent trials abolished the conflict-elicited evaluation effect. The results of Experiment 2 are also in line with the study of Fritz et al. (2015) who showed that conflict adaptation was selectively present in fluent but not in non-fluent conditions.

In Experiment 2a, we chose to increase the frequency of the incongruent conditions although we could have also increased the frequency of the congruent trials to try and further boost conflict-elicited evaluations. In the current experiment, we did not do so, as most prior studies used already increased frequencies of congruent conditions. For example, in the present Experiment 1, each congruent condition was four times as likely as each incongruent condition. Thus, we chose to vary the frequency of the incongruent trials, as we were not sure if a further increase in the frequency of the congruent conditions would have further increased the conflict-elicited evaluation.

## Experiment 2B

What remains to be studied after Experiment 2a is whether increased frequencies of specific incongruent conditions could have abolished the priming of a negative evaluation. This was tested in Experiment 2b. Here, we used only two Stroop colors and color word meanings. This increases the frequency of each specific incongruent stimulus, even while congruent and incongruent trials were equalized for their frequencies (in 50% of all trials). If the repetition of specific conflicting trials fosters processing fluency, again, a drop in the priming of negative evaluations of otherwise neutral stimuli was expected.

### Materials and Methods

#### Participants

Twenty-four new participants were tested. These participants did not take part in Experiment 2a. The sample contained 22 females and 2 males, *M*_*Age*_: 20.67 years, *SD*_*Age*_: 2.30 years.

#### Apparatus, Stimuli, and Procedure

Experiment 2b was similar to Fritz and Dreisbach (2013) with the exception that we only utilized two colors, for example, red and green, to create our congruent and incongruent Stroop stimuli. By doing so, we could equalize the number of congruent and incongruent trials, as well as the number of specific congruent and incongruent stimuli: Since we only used two different colors, there were only two possibilities for congruent (RED in red, GREEN in green) and incongruent signals (RED in green, GREEN in red). The colors used in Experiment 2b were counterbalanced between participants.

### Results

#### Evaluations

The mean ER was 2.23%. Catch trials and all trials with an incorrect reaction were omitted from the analysis. To test for a general bias toward positive or negative evaluations, we tested the percentages of negative judgments (across conditions) to the null hypothesis of 50%. We found no overall tendency (53.05% positive vs. 46.95% negative evaluations), *t*(23) = 1.89, *p* = 0.071, *d* = 0.39, BF = 1.01 in favor of the null hypothesis. We fitted the evaluations to a generalized linear mixed model with sensory congruency as a fixed factor. By-participants random intercepts and slopes for sensory congruency, as well as random intercepts by Chinese characters were used. There was a significant effect of sensory congruency, χ^2^(1) = 9.50, *p* = 0.002, indicating that the evaluations were significantly more often negative in the incongruent (estimate = −0.60, *SE* = 0.29) than in the congruent condition (estimate = 0.88, *SE* = 0.20). See also Table 1, lower row.

#### Reaction Times – Evaluation

Again, the time it took participants to rate the Chinese characters were centered and subjected to a linear mixed model, with evaluation (positive/negative) and sensory congruency (congruent/incongruent) as fixed effects and the same random effects structure as in Experiment 1a. No significant effects were found, all *F* < 0.41, all *p* > 0.249.

### Discussion

Experiment 2a left open the question if the overall higher frequency of conflicting than congruent trials or the higher frequency of each specific conflicting condition was responsible for weaker priming of negative evaluations. The results of Experiment 2b suggested that the mere increase of the frequency of each specific conflicting stimulus is not enough to eliminate the priming of a negative evaluation.

## Experiment 3

In Experiment 2a, we had seen that increasing the frequency of incongruent trials diminished the conflict-elicited priming of a negative evaluation of a neutral object. In Experiment 2b, we had tested and confirmed that this was not due to the increased frequency of specific sensory incongruent conditions.

However, what is unclear following Experiment 2a is if a lower frequency of congruent trials could have likewise diminished the degree of conflict and conflict-elicited evaluations. To note, in Experiment 2a, an increased frequency of the conflicting trials and a decreased frequency of the congruent trials were fully confounded: 75% conflicting trials in Experiment 2a meant that only 25% of trials were congruent. Importantly, a lower frequency of congruent trials alone could have invited less processing of the irrelevant word meaning (cf. Logan and Zbrodoff, 1979). This, in turn, would have decreased interference in conflicting trials and means that the lower frequencies of congruent trials alone could have diminished conflict-elicited priming of negative evaluations.

In Experiment 3, we tested if a lower frequency of congruent trials alone could diminish conflict-elicited priming of negative evaluations. To this end, we manipulated Stroop motor congruency. We used three types of conditions: motor congruent, motor neutral (or response-irrelevant), and motor conflicting trials. In this way, we were able to selectively decrease the frequency of the motor congruent trials without also having to increase the frequency of motor conflicting trials. In detail, by using altogether four color words (RED, BLUE, YELLOW, and GREEN) and only two colors (e.g., green and blue) to which participants had to respond, we created a novel motor neutral or response-irrelevant condition (e.g., the word RED in green) in addition to the motor congruent (e.g., the word GREEN in green) and the motor conflicting (e.g., the word BLUE in green) conditions. In this way, we were able to unconfound the frequency manipulations of the congruent and of the conflicting trials: By combining each of the four color words equally often with each of the two word colors, we created 25% motor congruent trials, 50% response-irrelevant trials, and 25% motor conflicting trials. As a result, in Experiment 3, we only decreased the frequency of the (motor) congruent trials (compared to Experiment 1b) but the frequency of the motor conflicting trials was not increased at the same time. This allowed us to check if the Stroop effect was diminished by the diminution of the frequency of the congruent trials.

### Materials and Methods

#### Participants

Twenty-four participants, 20 females and 4 males, *M*_*Age*_: 20.79 years, *SD*_*Age*_: 2.64 years, were tested.

#### Apparatus, Stimuli, and Procedure

Experiment 3 was similar to Experiment 1b. Participants had to classify the Stroop stimulus according to its print-color. Notably, only the print-colors blue and green (or red and yellow, counterbalanced across participants) were used in this experiment but all four color words were used. This manipulation allowed us to create sensory incongruent/response-irrelevant Stroop stimuli while still being able to ask the participants for their overt responses to the Stroop stimuli. Each print-color was mapped on one of two response keys. The mapping was counterbalanced across participants.

### Results

#### Reaction Times – Stroop Classification

We removed all trials with erroneous responses (5.68%) in the Stroop classification task and all trials with an RT <100 ms or >2.5 *SD*s of the mean RT of the individual participant per condition (3.44%). We fitted the centered correct RT to a linear mixed model with the same fixed and random effects structure as in Experiment 1b. A significant effect of congruency, *F*(2,28.11) = 8.24, *p* = 0.002, was followed up with pairwise Holm-corrected comparisons of the estimated marginal means of the model. These yielded a significant difference between motor-relevant/sensory congruent and response-irrelevant/sensory incongruent trials, *t* = -4.05, *p* < 0.001, but not for any other pairs, all *t* < |1.87|, all *p* > 0.148. See also Table 4.

**TABLE 4:** Reaction times (in ms) of the Stroop task as a function of sensory congruency (columns) and motor-relevance/response-irrelevance (rows) in Experiment 3.

	**Sensory congruency**	
	**Congruent (ms)**	**Incongruent (ms)**
Motor-relevant	553	616
Response-irrelevant	–	591

#### Error Rates – Stroop Classification

Error rates were subjected to a generalized linear mixed model similar to the RT and analogous to Experiment 1b. Contrasts were set to overall mean ER across all levels of the factor congruency. Participants made less errors (in comparison to the overall mean) in motor-relevant/sensory incongruent trials (estimate = 0.27, *SE* = 0.13, *z* = 2.13, *p* = 0.033) and more errors in response-irrelevant/sensory incongruent trials (estimate = −0.48, *SE* = 0.16, *z* = −3.07, *p* < 0.001).

#### Evaluations

The analysis was based on all data with a correct response in the Stroop classification task. We found an overall tendency for positive evaluations (55.84% positive vs. 44.16% negative evaluations), *t*(23) = 2.70, *p* = 0.013, *d* = 0.78. The results are summarized in Table 5. We fitted the evaluations to a generalized linear mixed model analogous to Experiment 1b. Contrasts were set to grand mean across all levels of the factor congruency. In relation to this overall mean (estimate = 0.24, *SE* = 0.09, *z* = 2.69, *p* = 0.007), none of the different levels of the factor congruency yielded significant differences, all estimates < |0.10|, all *z* < |1.56|, all *p* > 0.118, BF = 5.18 in favor of the null hypothesis.

**TABLE 5:** The percentages of negative evaluations as a function of sensory congruency (columns) and motor-relevance/response-irrelevance (rows) in Experiment 3.

	**Sensory congruency**	
	**Congruent (%)**	**Incongruent (%)**
Motor-relevant	45.62	44.75
Response-irrelevant	–	42.48

#### Reaction Times – Evaluation

As in the previous experiments, we subjected the centered evaluation times to a linear mixed model with the fixed factors congruency and evaluation (positive/negative) and their interaction. Random intercepts and slopes for this interaction by participant, as well as random intercepts by Chinese characters were used. No significant result was found, all *F* < 2.30, all *p* > 0.130.

#### Key Repetitions

Since the classification of the Stroop and the evaluation of the Chinese character were mapped on the same keys, we also checked whether participants simply pressed the same key twice. The proportion of trials where participants did this was not significantly different from 50%, *t*(23) = 0.31, *p* < 0.249, *d* = 0.09.

### Discussion

The results of Experiment 3 showed that decreasing the number of motor-congruent trials alone diminished the conflict-elicited priming of a negative evaluation of the otherwise neutral character. This is in line with the assumption that a lower number of congruent trials invites less usage of the irrelevant word meanings and, in turn, decreases the Stroop effect. This can be seen by comparing the motor-congruence Stroop effect in Experiment 1b with that in Experiment 3: There was a significant RT difference between sensory congruent/motor congruent and sensory incongruent/motor incongruent conditions in Experiment 1b. However, using a higher average frequency of motor-congruent trials, this RT congruence effect was no longer significant in Experiment 3. In line with an origin of the priming of negative evaluations in this type of motor conflict, the priming of negative evaluations was also no longer significant in Experiment 3.

However, to our surprise there was still a significant RT difference between sensory congruent/motor-relevant and sensory incongruent/response-irrelevant conditions. In fact, the response-irrelevant conditions created the slowest mean correct RTs. This effect, however, was obviously not of the same type as the typical Stroop conflict. This can be concluded from two observations. First, mere sensory incongruence between word color meaning and word print color created much less interference in Experiment 1b (34 ms delay relative to the sensory congruent/motor congruent conditions; Table 2) where the same conditions were motor-congruent than in Experiment 3 where these conditions were response-irrelevant (63 ms delay relative to the sensory incongruent/response-irrelevant conditions; Table 5). Second, whereas there was thus a much higher RT delay in Experiment 3, there was no priming effect of this delay on the negative evaluation of a neutral stimulus. This indicates that the corresponding RT delay probably did not reflect any kind of conflict. Instead, it probably reflected facilitation by all task-relevant word meanings, be they now motor-congruent or -incongruent.

## General Discussion

The current study is concerned with the potential origins of the priming of negative evaluations of otherwise neutral stimuli by preceding conflict. Past studies have demonstrated that prior conflict can elicit such priming of negative evaluations (e.g., Dreisbach and Fischer, 2012; Fritz and Dreisbach, 2013, 2015; Schouppe et al., 2015; Pan et al., 2016; Goller et al., 2017). However, the roles of a number of potential mediators of these influences were left open in prior studies, and some of these moderating influences were therefore studied in the present investigation. As these moderating influences were derived from existing theories and based on prior findings concerning the degree of interference in conflict tasks, their influences also indirectly support the interpretation of the priming of negative evaluations as being elicited by conflict.

In detail, we studied the role of overt responses to Stroop stimuli prior to the evaluations of subsequently presented neutral characters. This was done, as opposing predictions could be derived concerning the influence of such overt responses on the evaluations. First, prior findings showed that successful responses to conflicting stimuli can be felt as positive by the participants (Schouppe et al., 2015). Therefore, it would have been possible that once such successful responses were given in-between conflicting Stroop stimulus and to-be-evaluated neutral object, the negative evaluations of the neutral objects were diminished or even eliminated under these conditions compared to conditions without such intervening responses. Second, alternatively, it is known that Stroop conflict increases for response-incongruent word meanings, implying that the degree of conflict between sensory word print color and word color meaning could also be emphasized by words’ response relevance (cf. Klein, 1964). When we compared Stroop conflict’s priming of negative evaluations of neutral objects between Experiments 1a and 1b within the same participants, the latter view was better supported in the sense that the priming of the negative evaluations was definitely significant and present under conditions where an overt response was given to the conflicting stimuli.

This raises the question about which factor could have been responsible for the differences between studies showing conflict-elicited priming of negative evaluations even where overt responses were given to the conflicting primes (Damen et al., 2018; the present study) and studies demonstrating the reversed effect, at least following correct responses to conflicting prime stimuli (Schouppe et al., 2015; Ivanchei et al., 2018). One obvious reason could be the representation of the error signal that is assumed to be responsible for the reversal of the conflict-elicited priming (cf. Silvetti et al., 2011; Schouppe et al., 2015). In the studies that showed conflict-elicited priming of positive evaluations, clearly positive and negative words (Schouppe et al., 2015) or pictures (Ivanchei et al., 2018) were used as targets. Arguably, these targets created a strong expectancy-based reward signal themselves. This commonality between priming stimulus and target could be critical for the reversal of the effect, for example, by allowing a stronger carry-over of the reward signal from one (priming) stimulus to the next (target) stimulus. In contrast, the studies that found conflict-elicited priming of negative evaluations even after overt responses to the conflicting primes all used neutral, affectively ambiguous targets for which the corresponding feedback signal would have been very weak (Damen et al., 2018; the current study). In fact, it was impossible to categorize the responses to the affective valences of the ambiguous targets into correct and incorrect responses. This situation corresponds more to a switch of the task set from prime to target. Maybe the representation of a feedback signal to the prime – including its “affective content” (e.g., as positive following a correct response to a conflicting prime) – could have fallen prey to this switch.

In the current study, we then went on to study the role of diverse frequency manipulations. In Experiment 2a, we increased the frequency of conflicting Stroop stimuli. This was done to see if this would diminish the degree of interference (cf. Logan and Zbrodoff, 1979), here measured by the priming of a negative evaluation only. This prediction was confirmed. However, Experiment 2a not only increased the overall frequency of incongruent conditions but also increased the frequency of each specific conflicting condition. Thereby the frequency of the congruent trials was also decreased. Furthermore, as each of these latter two influences could have likewise diminished the priming of negative evaluations, these possibilities were tested in turn in Experiments 2b and 3. On the one hand, Experiment 2b showed that the higher frequency of each particular conflicting condition in and by itself does not abolish the priming of a negative evaluation. On the other hand, Experiment 3 showed that the reduction of motor-congruent trials alone could have had a similar diminishing impact on conflict-elicited negative evaluations. This impact is similar to the concomitant overall increase of frequencies of incongruent conditions plus the decrease of frequencies of congruent conditions in Experiment 2a. Admittedly, in Experiment 3 this diminution of the priming of negative evaluations was found where participants gave an overt response to the conflicting stimuli and prior to the evaluations. However, as Experiment 1b confirmed that giving such an overt response in itself does not prevent the priming of negative evaluations, it is unlikely that the overt responses in Experiment 3 were responsible for the lack of priming of negative evaluations.

### Limitations of the Current Study

One limitation is that a Stroop effect in overt responses was not measured in all of the experiments. For example, in Experiment 2, we assumed that our manipulations affected the degree of conflict and, thereby, the priming of negative evaluations, but as no Stroop effect was measured with the help of overt responses, the success of our manipulation of the degree of conflict is in doubt. In addition, once we measured overt correct RTs, at least the slow responses to response-irrelevant Stroop stimuli in Experiment 3 were surprising. We speculated that these “delays” were actual reflections of facilitation by all task-relevant color word meanings, but as long as there is no independent measurement or manipulation confirming this assumption, this remains a speculation. Furthermore, another weakness of Experiment 3 was that for our test of the influence of lowered frequencies of congruent trials on the size of the Stroop effect and on the conflict-elicited priming of a negative evaluation, we had to use motor congruency. This was necessary to keep the frequency of the corresponding incongruent condition, here: the response-irrelevant condition, equally low and, thus, to dissolve the confounding influences of higher frequencies of incongruent and lower frequencies of congruent conditions that existed in Experiment 2a. However, this meant that we had to compare motor congruence effects in Experiment 3 to mere sensory congruence effects in Experiment 2a. As motor congruence could modify the amount of sensory congruence (for evidence, see, e.g., the lower interference by sensory incongruent/motor congruent conditions in Experiment 2b), this comparison of frequency influences of congruent trials across Experiments 2a and 3 is relatively indirect. A final shortcoming of the present study is that not all our manipulations were conducted as within-participant variables. Some were also realized by between-participants variables, such as the comparison between Experiments 2a and 3. As the differences in the results could thus have also reflected chance differences between participants, not all of our findings are equally certainly due to the manipulations of the independent variables. Some might also be due to between-participants differences alone.

## Conclusion

In the present study, we have once more replicated the important conflict-elicited tendency to evaluate otherwise neutral objects as more negative by Fritz and Dreisbach (2013), and we have also identified critical side conditions for this effect. In particular, we showed that overall frequencies of congruent and incongruent trials could diminish the effect; that the overt responses to the conflicting stimuli did not abolish the effects; and that assigning response congruence status to sensory incongruent Stroop conditions had also a strong modulating role on conflict-elicited priming of negative evaluations.

## Data Availability Statement

The datasets generated for this study are available on request to the corresponding author.

## Ethics Statement

All studies were conducted in accordance with the Declaration of Helsinki (revised, 1983) and the guidelines of the Faculty of Psychology, University of Vienna, Vienna, Austria. We further followed the Austrian Universities Act, 2002 (UG2002) – which was active at the time of the experiments – which required only medical universities to appoint ethics committees for clinical testing, and application of medical methods and applied medical research. Therefore, no additional ethical approval was sought.

## Author Contributions

FG and UA developed the study concept and the study design. FG performed the data collection, data analysis, and interpretation under the supervision of UA. FG drafted the manuscript, both AK and UA substantially contributed to the interpretation of the data and provided many important critical revisions. All authors approved the final version of the manuscript for submission and agreed to be accountable for all aspects of the work in ensuring that questions related to the accuracy or integrity of any part of the work are appropriately investigated and resolved.

## Conflict of Interest

The authors declare that the research was conducted in the absence of any commercial or financial relationships that could be construed as a potential conflict of interest.

## Footnotes

1 http://www.mdbg.net/chindict/chindict.php
